# Enhancing problem-solving skills and creative thinking abilities in U-13 soccer players: the impact of rondo possession games’ training

**DOI:** 10.5114/biolsport.2025.146782

**Published:** 2025-03-18

**Authors:** Mohamed Mansour Bouzouraa, Wissem Dhahbi, Hatem Ghouili, Jaouher Hamaidi, Mohamed Ben Aissa, Ismail Dergaa, Noomen Guelmami, Nizar Souissi, Katja Weiss, Thomas Rosemann, Makrem Zghibi, Karim Chamari, Beat Knechtle

**Affiliations:** 1 High Institute of Sport and Physical Education of Ksar Said, University of Manouba, Manouba, Tunisia; 2 Research Unit “Sport Sciences, Health and Movement”, Higher Institute of Sports and Physical Education of Kef, University of Jendouba, El Kef, Tunisia; 3 Qatar Police Academy, Police College, Training Department, Doha, Qatar; 4 Research Unit Physical Activity, Sport, and Health, UR18JS01, National Observatory of Sport, Tunis, Tunisia; 5 Primary Health Care Corporation (PHCC), Doha, Qatar; 6 Research Laboratory Education, Motricité, Sport et Santé (EM2S) LR19JS01, High Institute of Sport and Physical Education of Sfax, University of Sfax, Sfax, Tunisia; 7 High Institute of Sport and Physical Education of El Kef, University of Jendouba, El Kef, Tunisia; 8 Postgraduate School of Public Health, Department of Health Sciences (DISSAL), University of Genoa, Genoa, Italy; 9 Institute of Primary Care, University of Zurich, Zurich, Switzerland; 10 Naufar, Wellness & Recovery Center, Doha, Qatar; 11 Medbase St. Gallen Am Vadianplatz, St. Gallen, Switzerland

**Keywords:** Adolescent, Football, Cognitive skills, Decision-making, Game intelligence, Soccer coaching, Youth sports

## Abstract

To investigate the impact of Rondo possession games, played in different field geometries with complex rules, on the development of problem-solving skills and creative thinking abilities in U-13 youth soccer players. In a repeated-measures and a randomized controlled trial study design, twenty-four competitive young male U-13 soccer players were split into: the training (TG, n = 12) and control (CG, n = 12) groups (matched for age, body height body mass index; and training experience; with weekly training of ~5.5 hours/week for both groups). TG performed a training programme once a week for 8 weeks integrating Rondo possession games with periodized variations, such as player formats, field shapes, duration, pitch sizes, and rest times. Torrance Tests of Creative Thinking (TTCT) – Graphic-Figural-Creativity (abstractness-title, resistance-closure, originality, elaboration, and fluency – GFC), Problem-Solving-Inventory (problem-solving-confidence, approach-avoidance-style and personal-control – PSI) and Verbal-Creativity (flexibility, originality, fluency – VC) – were performed over time and between groups. We observed significant main effects of time (p < 0.001–0.005, *ƞ*^2^p: 0.30–0.76 [large]) and group (p < 0.001–0.002, *ƞ*^2^p: 0.36–0.60 [large]), as well as a significant interaction effect of time × group (p: < 0.001–0.013, *ƞ*^2^p: 0.24–0.48 [large]) of all GFC, PSI and VC test indices. In the TG group, all TTCT indices showed significant improvement in all indices at the post-test compared to pre-test (-29.90%-227.27%, d: 1.03–4.66 [large]). This study showed that 8 weeks of integrated Rondo possession games with structured geometric variations and rule complexity significantly boosted problem-solving skills and creative thinking in U-13 soccer players. These findings suggest a promising direction for youth soccer training, focusing on developing well-rounded players capable of intelligent and flexible gameplay in diverse scenarios.

## INTRODUCTION

Soccer is one of the most popular sports worldwide, offering an excellent avenue for players [1] to engage in healthy intensive physical activity [2–4]. Soccer also promotes various mental and social skills such as problem-solving skills and creative thinking abilities [5, 6]. Different methodologies and game formats are employed within soccer training to enhance specific aspects of player development, such as Rondo drills [7]. Rondo games (RGs) have emerged as a cornerstone training method in soccer. They are meticulously designed to refine critical skills directly relevant to real-world competition: ball retention, passing accuracy, and rapid decision-making under pressure [8–11] from the opponents. RGs demonstrate high adaptability, allowing the difficulty level to be readily adjusted to cater to individual player skill levels and specific training objectives. Additionally, variations in formation, player numbers, and space constraints can be introduced to further mirror the dynamic and unpredictable nature of soccer matches [11]. This dynamic setup fosters not only skilful execution but also vital tactical awareness, including effective communication, seamless teamwork, and heightened temporal aspects in addition to the spatial recognition on the field [12].

In the dynamic world of soccer, effective problem solving lies at the heart of on-field success. This necessitates a nuanced interplay of mental agility, spatial awareness, and seamless communication [13]. RGs provide a fertile training ground for cultivating these crucial skills [14]. RGs inherent adaptability, manifest in variable shapes and rule sets, compels players to constantly evaluate game dynamics (i.e. scanning ability) and adjust their strategies accordingly [15]. This iterative process fosters enhanced problem-solving capabilities, as players refine their ability to analyse situations, make rapid tactical decisions, and develop creative solutions under time pressure. Moreover, the fast-paced nature of RGs requires rapid cognitive processing, further honing players’ problem-solving skills and decision-making process [16]. Beyond individual benefits, RGs with intricate shapes and complex rules foster team cohesion and overall performance by encouraging adaptation, anticipation, and collaborative problem solving [16].

A research gap exists in understanding the impact of RGs on the crucial yet understudied cognitive skills of problem solving and creative thinking in soccer players. While existing research acknowledges the effectiveness of possession games in enhancing technical and tactical skills [17, 18], their influence on cognitive development remains largely unexplored. This gap is particularly important in youth player development, where problem solving is considered a key facet of creative thinking and players’ development [19–21] Despite this recognition, empirical studies testing the effectiveness of existing problem solving and creative thinking tests with young players are scarce [22, 23]. Investigating the effects of RGs on these cognitive skills is crucial for several reasons. First, it holds practical implications for coaches by offering a deliberate practice that integrates diverse skills beyond technical mastery. These skills, including adaptability, quick thinking, and innovative problem solving, are highly transferable to various real-life situations. Second, understanding the impact of RGs on cognitive development can inform and optimize training design. By providing insights into how RGs influences problem solving and creative thinking, we intend to expand the training model and encourage the integration of RGs.

The developmental phase of young soccer players is critical for acquiring key soccer-and everyday life-related skills [24]. Rondo possession games present an ideal platform for enhancing cognitive abilities. Therefore, we aimed to simulate various scenarios in Rondo possession games with varied geometric shapes and complex rules, to investigate the potential improvement of problem-solving skills and creative thinking in young soccer players.

## MATERIALS AND METHODS

### Participants

G*Power software (version 3.1.9.6; Kiel University, Kiel, Germany) was used to calculate the minimum required sample size. A sample size of 24 participants would be sufficient to detect significant differences (effect size *f* = 0.65, α = 0.05) with an actual power of 80%. Twenty-four competitive young male U-13 soccer players, playing in an elite soccer academy and regional league of the regional team-soccer championship, were randomly assigned to the training (TG, n = 12) and the control (CG, n = 12) groups (age: 11.92 ± 0.67 and 12.08 ± 0.67 years; height: 156.25 ± 5.88 and 155.17 ± 3.13 cm; body mass index (BMI): 15.9 ± 3.2 and 14.9 ± 1.8 kg · m^−2^; training experience: 4.17 ± 1.03 and 4.42 ± 0.90 years, respectively; weekly training: ~5.5 hour/week for both groups). During the experimental period, soccer training sessions of ~1 h 15 min, 4 times per week, were simultaneously performed by both groups, in addition to one weekly official match. The study inclusion criteria were: no moderate or severe injuries within the 1 month preceding the study. Before the study began, all participants completed and signed written informed consent forms. This exclusion criterion was crucial to ensure that participants did not have any conditions that could potentially bias the results or hinder their ability to accurately perform the required physical tasks. All participants were informed of the intent and procedures of the study and signed an informed consent form before data collection. Written parental consent was obtained for all participants. The research project underwent a comprehensive review and received approval for implementation from the local Personal Protection Committee (PPC: No. C-041-2023), and was conducted in accordance with the recommendations of the World Health Organization outlined in the Declaration of Helsinki.

### Study design

A repeated-measures experimental design with a randomized controlled trial was used. In order to assess the effects of the programme training of Rondo possession games, the Graphic-Figural-Creativity (abstractness-title, resistance-closure, originality, elaboration and fluency – GFC), Problem-Solving-Inventory (problem-solving-confidence, approach-avoidance-style and personal-control – PSI) and Verbal-Creativity (flexibility, originality, fluency – VC) test procedures were conducted before and after an experimental training period of eight weeks. For the TG, the experimentations integrated in their regular training programme a specific exercise (e.g., Rondo possession games with periodized different variations). The CG followed its usual fitness and traditional technical-tactical soccer training programme without RGs (passing, dribbling shooting, defending and teamwork exercises, i.e., the same training load as TG).

### Procedure

**FIG. 1:**
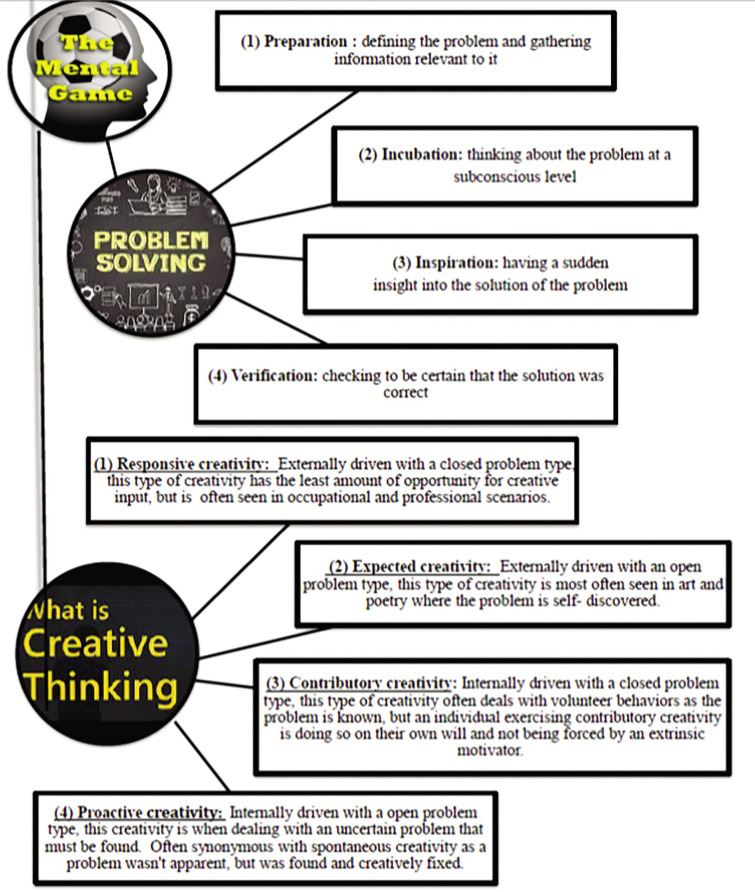
Problem-Solving Skills and Creative Thinking Ability Theories

Pre-test assessments comprised (i) anthropometric characteristics: body height, body mass, BMI and (ii) problem-solving skills through the utilization of the PSI. The Torrance Tests of Creative Thinking (TTCT) assessed the creative thinking abilities of the participants. They measure divergent thinking skills, fluency, flexibility, originality, and elaboration (Figure 1). Participants were tasked with generating multiple solutions, thinking innovatively, and demonstrating creativity in their responses. The training programme consisted of eight sessions of Rondo Possession Games, conducted over a two-month period (eight weeks), with one session per week. These games are designed with various shapes and formats to provide participants with both variety and challenge.

### Rondo possession games with complex rules training programme

The Rondo Possession Games were structured as follows: Session 1: Rondo in triangle shape (7 vs. 5); Session 2: Rondo in hexagon shape (3 vs. 3+6); Session 3: Rondo in rhombus shape (8 vs. 4); Session 4: Rondo in circle shape (4 vs. 4+4); Session 5: Rondo in square with 1 zone (5 vs. 5+2); Session 6: Rondo in square with 2 zones (4 vs. 3 to 4 vs. 1); Session 7: Rondo in square with 3 zones (4 vs. 2); Session 8: Rondo in square with 4 zones (1 vs. 1+4). The periodization of training load sessions, including player formats, field shapes, duration, pitch sizes [7], and rest time, is effectively demonstrated in Table 1 and Table 2

**TABLE 1:** Rondo Possession Games (RGs) in different conditions

RGs	Format	Shapes	Duration	Spaces / Pitch Sizes	Rest	Approximate workload
Game 1	7 v 5	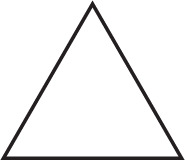	3 min	15 m × 15 m	2 min	6 repetitions

Game 2	3 v 3+6	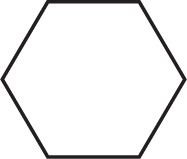	5 min	25 m × 25 m	1 min	5 repetitions

Game 3	8 v 4	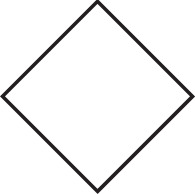	2 min	13 m × 13 m	1 min	8 repetitions

Game 4	4 v 4+4	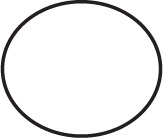	6 min	Diameter = 35 mRayon = 17.5 m	2 min	3 repetitions

Game 5	5 v 5+2	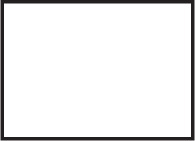	4 min	30 m × 30 m	1 min	4 repetitions

Game 6	4v3 to 4 v 1	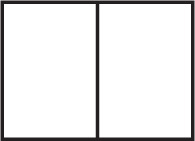	3 min	18 m × 18 m Within each zone	1 min	5 repetitions

Game 7	4 v 2	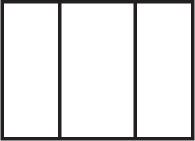	4 min	Zone 1 and 3; 20 m × 20 mZone 2; 5 m × 20 m	2 min	4 repetitions

Game 8	1 v 1+4	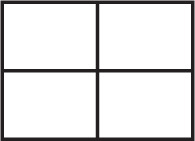	4 min	12 m × 12 m. Within each zone	1 min	4 repetitions

**TABLE 2:** Description of Complex Rules during Rondo Possession Games

**Rondo Game 7 v 5**. **Team Composition**: Red team consists of 7 players, 3 positioned outside the triangular area and 4 within it. Blue team comprises 5 players positioned solely within the triangle. **Playing Area**: A triangular zone with 3 small goals located at each corner **Scoring System:** Red team: 1 point for three consecutive passes within the triangle, resulting in a goal at one of the triangle’s corners. 2 points for six passes within the triangle, involving outside players, followed by a goal. Blue team: 1 point for intercepting the ball and scoring directly into the goal. 3 points for initiating a pass exchange with an outside Red player before scoring.	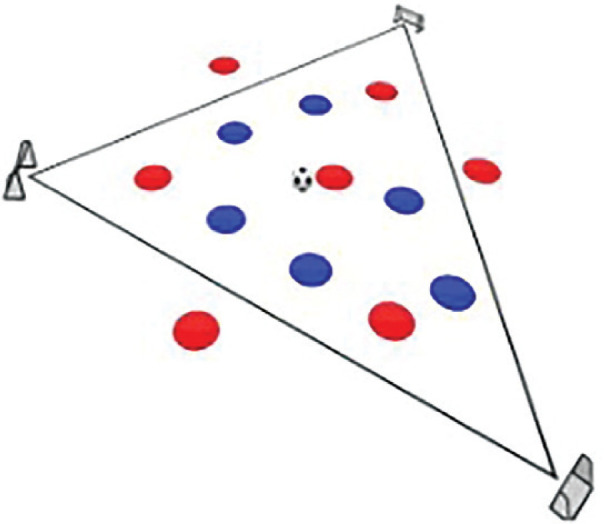

**Rondo Game 3 v 3 + 6**. **Team Composition**: Red team: 3 players within a hexagon. Black team: 3 players within a hexagon. Yellow team: 3 players outside the hexagon. Blue team: 3 players outside the hexagon. **Playing Area:** A hexagon with 6 small goals located at each corner. **Scoring System:** Red team: 1 point for scoring a goal in any of the six corner goals within the hexagon. 2 points for executing 10 successful passes between the Red team and the Yellow/Blue teams. Black team: 4 points for intercepting the ball and scoring a goal.	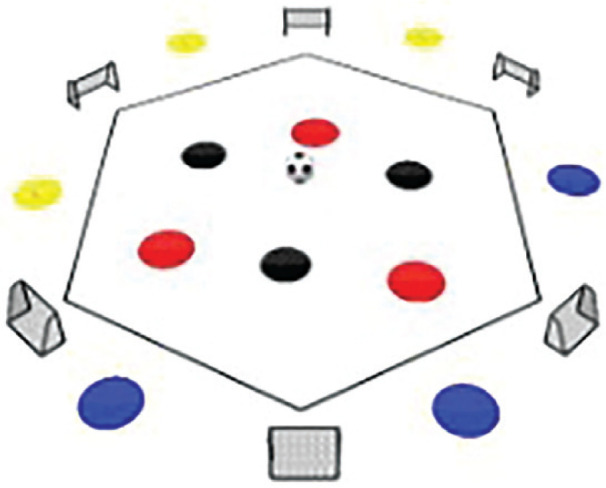

**Rondo Game 8 v 4. Team Composition:** Red team: 4 players within a rhombus. Blue team: 4 players within a rhombus and 4 players positioned outside the rhombus. **Playing Area:** A rhombus with 4 small goals located at each corner. **Scoring System:** Red team: 2 points for completing 5 passes with one touch and scoring a goal. 1 point for completing 10 passes with two touches and scoring a goal. Blue team: 3 points for intercepting the ball from the Red team and scoring a goal with all team members touching the ball.	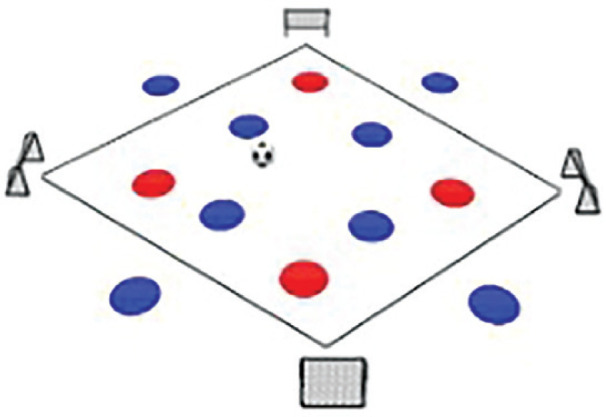

**Rondo Game 4 v 4 + 4**. **Team Composition:** Red team: 4 players within a circle. Blue team: 4 players within a circle. Yellow team: 4 players positioned outside the circle. **Playing Area:** A circle with 4 small goals distributed throughout its circumference, each uniquely identified by numbered and color-coded cones. **Scoring System**: Red team: 4 points for completing 10 passes with the Yellow team players positioned outside the circle within 20 seconds and scoring a goal in one of the small goals. Blue team: 3 points for intercepting the ball from the Red team and scoring in goal 1. 4 points for intercepting the ball from the Red team and scoring in goal 2. 1 point for intercepting the ball from the Red team and scoring in goal 3. 2 points for intercepting the ball from the Red team and scoring in goal 4.	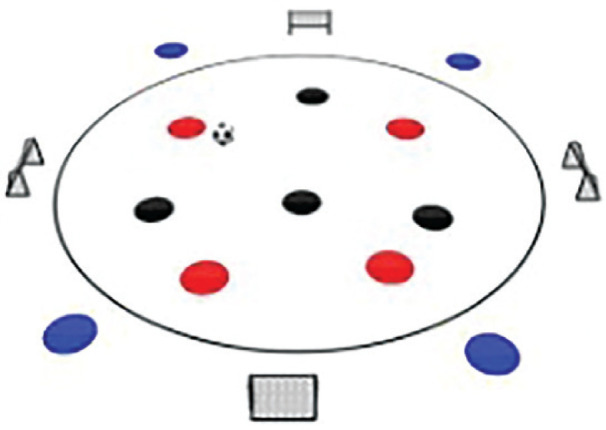

**Rondo Game 5 v 5 + 2. Team Composition:** Red team: 5 players within a square. Blue team: 5 players within a square. Neutral players Yellow team: 2 players positioned outside the square. **Playing Area:** A square with 8 goals distributed along its sides. **Scoring System:** Blue team: 2 points for exchanging passes and manoeuvring the ball between the neutral players (1 and 2) a total of 4 times and scoring a goal, with the pivotal pass originating from one of the neutral players. Red team: 2 points for intercepting the ball and scoring a goal. 3 points for one of the neutral players scoring a goal for the Red team.	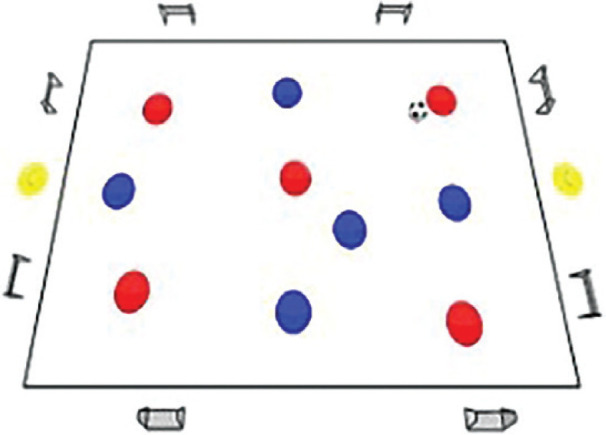

**Rondo Game 4 v 3 to 4 v 1. Team Composition:** Blue team: 4 players in one half of the square and 0 players in the other half. Black team: 3 players in one half of the square and 1 player in the other half. Red team: 0 players in one half of the square and 4 players in the other half. **Playing Area:** A square with 2 small goals, one in each half. **Scoring System:** Blue team: 2 points for executing 10 passes with the Red team in the second half within a time frame of 25 seconds and scoring a goal. Black team: 3 points for intercepting the ball from the Blue team in the first half and completing three passes to score a goal. 5 points for the Black team player in the second half intercepting the ball from the Red team and scoring a goal.	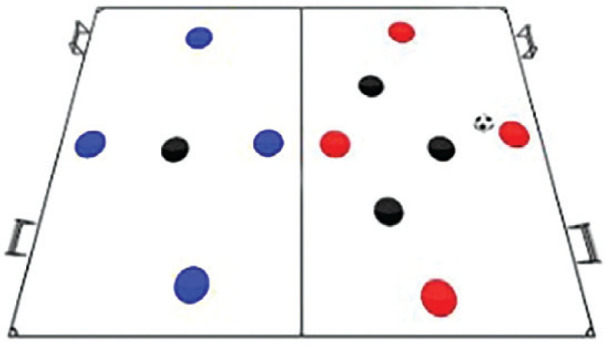

**Rondo Game 4 v 2. Team Composition:** Blue team: 4 players in the first area of the square and 4 players in the second area of the square. Black team: 2 players in the first area of the square, 2 players in the central zone of the square, Red team: 4 players in the second area of the square. **Playing Area:** A square with 2 small goals, one in the first area and one in the second area. **Scoring System:** Blue team: 1 point for completing 15 passes between the first and second areas. Black team: 2 points for intercepting the ball from the Blue team in the first area and scoring a goal, with the scoring player dribbling past a Blue team player. 2 points for intercepting the ball from the Blue team in the central zone and scoring a goal in the second area.	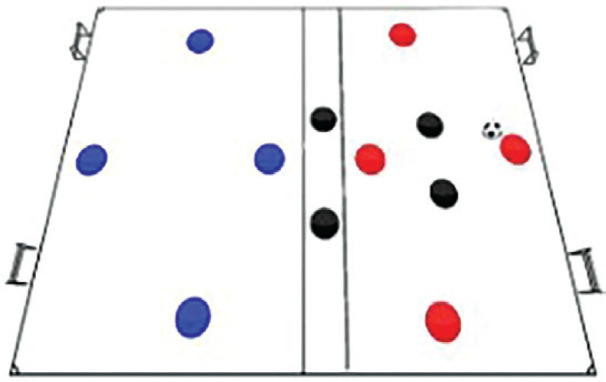

**Rondo Game 1 v 1 + 4. Team Composition:** Red team: 4 players, each positioned in one of the four areas of the square. Blue team: 4 players positioned outside the square. Black team: 4 players, each positioned in one of the 4 areas of the square. **Playing Area**: A square divided into 4 distinct areas, each featuring a 1 small goal. **Scoring System:** Red team: 2 points for successfully exchanging passes and manoeuvring the ball through all 4 zones before scoring in the goal. 5 points for navigating the ball across all zones during a passing exchange involving the four neutral players. Blue team: 1 point for intercepting the ball and scoring directly without passing it through the zones. 2 points for passing the intercepted ball from one area to the adjacent area. 4 points for passing the intercepted ball from the intercepted area to the next area via a diagonal pass after scoring a goal.	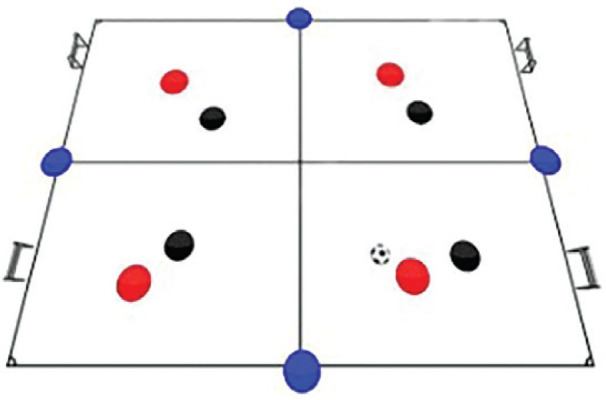

### Testing procedures

***Torrance Tests of Creative Thinking:*** In order to assess the effects of the programme training of Rondo possession games in both the pretest and post-test phases, we administered the Torrance Tests of Creative Thinking (TTCT [25]). The TTCT-Verbal and the TTCT-Figural each have two distinct variants.

The Arabic version of the TTCT-Verbal test encompasses six distinct activities designed to evaluate various aspects of creative thinking [26]. The first activity, ‘Asking,’ involved participants listing questions related to a depicted image (5 minutes). This was followed by ‘Guessing Causes,’ where subjects speculated on possible causes for the action, and ‘Guessing Consequences’ (5 minutes), focusing on potential outcomes of the action. The fourth activity, ‘Product Improvement,’ required participants to suggest improvements to a toy monkey (10 minutes). In the fifth activity, ‘Unusual Uses’ (10 minutes), participants were required to devise novel uses for tin cans. The final activity, ‘Just Suppose,’ encouraged participants to contemplate the implications of an unlikely scenario (5 minutes). The assessment criteria for these responses included fluency, gauged by the number of relevant responses generated; flexibility, determined by the variety of categories reflected in the responses as per the flexibility categories in the scoring manual; and originality, quantified based on response frequencies, with two points awarded for responses less common than 2% in the sample, one point for those between 2% and 5%, and no points for responses more prevalent than 5% [27]. In the graphic-figural section, three 10-minute activities test fluency, originality, abstractness of title, resistance to premature closure, and elaboration. Fluency is measured by the number of ideas generated in the second and third activities, with each idea earning a point. Originality follows the same scoring as in the verbal tasks. The abstractness of the title, tested in the first activity, is scored based on the degree of abstractness, with up to three points possible. Resistance to premature closure, evaluated in the second activity, rewards less direct or quick figure completions with higher scores. Finally, elaboration is scored by the number of additional details provided in the first and second activities, encouraging the development and extension of ideas (Table 3) [28]. The three activities allowed evaluation of several aspects of creative thinking, five of which were measured in the present study, as shown in Table 3.

**TABLE 3:** Evaluation of Creative Thinking Aspects through Three Activities

Activity	Description	Scoring Criteria for Creative Thinking
**Construction of a drawing**	Participants create a drawing using a black blot and give it a title.	**Originality**: Assessed based on the idea’s statistical frequency. **Abstractness of the Title**: Scored from 0 to 3 based on the title’s abstractness.

**Drawings to Complete**	Participants complete given incomplete figures.	**Fluency**: 1 point for each idea produced. **Resistance to Premature Closure**: Scored from 0 to 3 based on the closure type; quicker and more direct closures receive lower scores. **Elaboration**: Scored from 0 to 3 based on the number of additional details beyond the basic idea.

**Lines/Circles**	Participants use parallel lines or circles to create drawings.	**Fluency**: As in ‘Drawings to Complete’ **Originality**: Assessed as in ‘Construction of a Drawing’. **Elaboration**: As in ‘Drawings to Complete’.

In terms of psychometrics, both formats demonstrate high reliability, with studies showing good consistency across diverse populations. Recent studies, particularly for the TTCT-Verbal, suggest a multifactorial structure, contributing to a better understanding of its construct validity. The TTCT-Figural shares similar challenges and advancements in validity as the Verbal version, with studies enhancing our understanding of its construct validity. Recent studies, including those focusing on Arabic-speaking populations, identified a general factor corresponding to specific activities of the test. This model displayed robust reliability and item fit, enhancing our understanding of creativity measurement.

***The Problem-Solving Inventory (PSI)*** The Arabic version of PSI was used to evaluate individuals’ perceptions of their problem-solving abilities and styles across three dimensions: Problem-Solving Confidence (11 items), Approach-Avoidance Style (16 items), and Personal Control (5 items). Each of the 35 questions is rated on a sixpoint scale, from 1 (Strongly Agree) to 6 (Strongly Disagree). Higher scores in Problem-Solving Confidence signify lower confidence levels in one’s problem-solving skills, while elevated scores in Approach-Avoidance Style suggest a tendency to avoid rather than confront problems. Similarly, higher scores in Personal Control reflect a more negative perception of personal control over encountered issues. The total PSI score, calculated by summing responses, serves as an overall measure, with lower scores indicating more effective problem-solving abilities. This tool provides valuable insights into individuals’ approaches to problem-solving, aiding in targeted interventions to enhance their skills based on perceived strengths and challenges [29].

### Statistical analysis

Data were analysed using SPSS Statistics version 28.0 for Windows (IBM Corp., USA). Means and standard deviations (SD) were calculated after verifying normality of distributions using the Shapiro–Wilk procedure. The data were analysed using a multifactorial two-way (Time [Test, Retest] × group [Trained, Control]) ANOVA. Greenhouse-Geisser corrections were used when the assumption of sphericity (Mauchly’s test) was violated. To help protect against type II errors, an estimate of effect size (*ƞ*^2^p) was calculated. Tukey-adjusted pairwise post-hoc comparisons were performed when appropriate. Effect sizes for pairwise comparisons were calculated as Cohen’s *d*. Significance level was set *a priori* at *p* < 0.05.

## RESULTS

Residual data for anthropometric characteristics and psychological test scores (i.e., verbal creativity, graphic-figural creativity and problem-solving inventory) were normally distributed (*p* = 0.062–0.220). The independent sample t-test revealed no difference between groups for age, body mass, height, weekly training volume, or training experience (*p*: 0.11–0.50; *d*: 0.10–0.48 [*trivial*-*small]*)

## Graphic-Figural Creativity

**FIG. 2:**
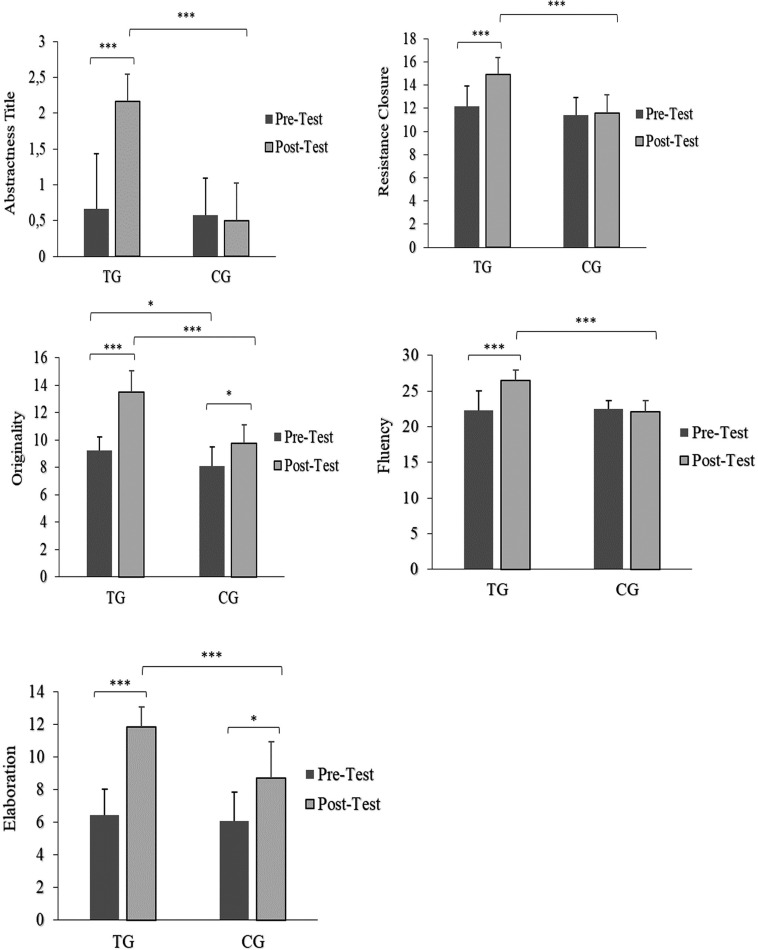
Graphic-Figural Creativity index values before and after training period. Footnote: TG = Training Group; CG = Control Group; Significance levels: *p < 0.05, ***p < 0.001

There was a significant main effect of time (*p*: < 0.001–0.005, *ƞ*^2^p: 0.30–0.76 [*large*]) and group (*p*: < 0.001–0.002, *ƞ*^2^p: 0.36 – 0.60 [*large*]), as well as a significant interaction effect of time × group (*p*: < 0.001–0.013, *ƞ*^2^p: 0.24 – 0.48 [*large*]) of all Graphic-Figural Creativity indices (i.e., abstractness title, resistance closure, originality, elaboration and fluency). In the TG, the pairwise comparisons showed a significant improvement of all Graphic-Figural Creativity indices at post-test compared to the pre-test. Specifically, the abstractness title index improved by 227% (*d* = 1.65 [*large*]), the resistance closure index improved by 22.6% (*d* = 1.03 [*large*]), the originality index improved by 45.9% (*d* = 2.75 [*large*]), the elaboration index improved by 83.7% (*d* = 3.74 [*large*]), and the fluency index improved by 19.1% (*d* = 1.29 [*large*]). On the other hand, in the CG, all Graphic-Figural Creativity indices showed non-significant differences in the pairwise comparisons at post-test compared to the pre-test (*p* > 0.05; *d*: 0.09–0.18 [*small*]), with the exception of the originality parameter, which showed a significant difference (20.66%; *p* < 0.05; *d* = 0.88 [*large*]) (Figure 2).

## Verbal Creativity

**FIG. 3:**
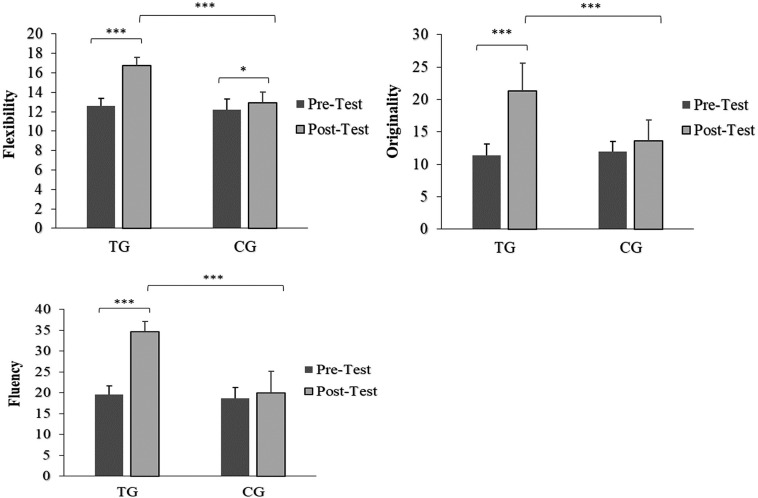
Verbal Creativity index values before and after training period. Footnote: TG = Training Group; CG = Control Group; Significance levels: *p < 0.05, ***p < 0.001.

There was a significant main effect of time (*p*: < 0.001, *ƞ*^2^p: 0.70 – 0.82 [*large*]) and group (*p*: < 0.001, *ƞ*^2^p: 0.42 – 0.68 [*large*]), as well as a significant interaction effect of time × group (*p*: < 0.001, *ƞ*^2^p: 0.55 – 0.69 [*large*]) of verbal creativity indexes (i.e., flexibility, originality, and fluency). In the training group (TG), the pairwise comparisons showed a significant increase of flexibility (32.9%; *d* = 3.97 [*large*]), originality (88.1%; *d* = 2.66 [*large*]), and fluency (77.2%; *d* = 3.09 [*large*]) at post-test compared to the pre-test. Additionally, at post-test, the analysis showed that for all verbal creativity indices, TG was significantly higher than the CG (*p* < 0.001). On the other hand, the CG also showed a significant improvement in the flexibility index from pre- to post-test (5.9%, *p* = 0.046, *d* = 0.55 [*moderate*]) (Figure 3).

## Problem-Solving Inventory

**FIG. 4:**
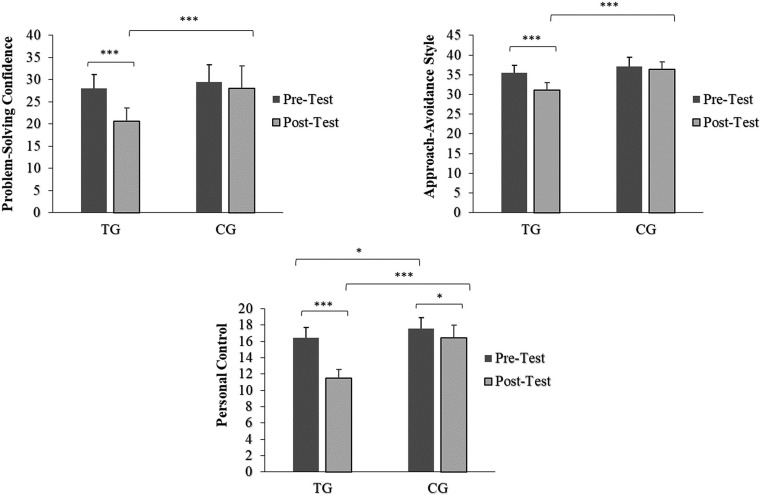
Problem-Solving Inventory index values before and after training period. Footnote: TG = Training Group; CG = Control Group; Significance levels: *p < 0.05, ***p < 0.001.

There was a significant main effect of time (*p*: < 0.001–0.002, *ƞ*^2^p: 0.37–0.67 [*large*]) and group (*p*: < 0.001, *ƞ*^2^p: 0.47–0.81 [*large*]), as well as a significant interaction effect of time × group (*p*: < 0.001–0.009, *ƞ*^2^p: 0.27 – 0.63 [*large*]) (Table 4). In the training group (TG), the pairwise comparisons showed a significant decrease of problem-solving confidence (-26.0%; *d* = 1.32 [large]), approach-avoidance style (-12.4%; *d* = 2.02 [*large*]), and personal control (-29.9%; *d* = 4.66 [*large*]) at post-test compared to the pre-test. Additionally, at post-test, the analysis showed that for all problem-solving inventory indices, TG was significantly higher than CG (*p* < 0.001). On the other hand, the CG also showed a significant decrease in the Personal Control index from pre to post-test (-6.4%, *p* = 0.015, *d* = 0.62 [*moderate*]) (Figure 4).

**TABLE 4:** Comparison of creative thinking abilities and problem-solving index values before and after training period between training group and control group

Parameters		Group	Change %	Cohen’s *d*	Main Effectp-value (ƞ_p_^2^)		Interaction Effect *p*-value (ƞ_p_^2^)

					Time	Group	Group * Time
**Graphic-Figural Creativity**	Abstractness Title	TG	227.27	1.65	< 0.001 (0.43)	< 0.001 (0.59)	< 0.001 (0.48)
		CG	-13.79	0.10			

	Resistance Closure	TG	22.61	1.03	0.005 (0.30)	< 0.001 (0.49)	0.011 (0.26)
		CG	1.48	0.09			

	Originality	TG	45.94	2.75	< .001 (0.76)	< 0.001 (0.60)	0.001 (0.38)
		CG	20.66	0.88			

	Elaboration	TG	83.72	3.74	< 0.001 (0.73)	0.002 (0.36)	0.013 (0.24)
		CG	43.02	0.80			

	Fluency	TG	19.13	1.29	0.002 (0.37)	< 0.001 (0.43)	< 0.001 (0.44)
		CG	-1.51	0.18			

**Verbal Creativity**	Flexibility	TG	32.93	3.97	< 0.001 (0.82)	< 0.001 (0.68)	< 0.001 (0.69)
		CG	5.99	0.55			

	Originality	TG	88.19	2.66	< 0.001 (0.70)	< 0.001 (0.42)	< 0.001 (0.55)
		CG	13.68	0.40			

	Fluency	TG	77.27	3.09	< 0.001 (0.74)	< .001 (0.77)	< 0.001 (0.67
		CG	7.56	0.20			

**Problem Solving**	Problem-Solving Confidence	TG	-26.06	1.32	0.002 (0.37)	< 0.001 (0.47)	0.008 (0.27)
		CG	-5.05	0.36			

	Approach-Avoidance Style	TG	-12.40	2.02	< 0.001 (0.43)	< 0.001 (0.66)	0.009 (0.27)
		CG	-2.12	0.20			

	Personal Control	TG	-29.90	4.66	< 0.001 (0.67)	< 0.001 (0.81)	< 0.001 (0.63)
		CG	-6.48	0.62			

Footnote: TG: Training Group; CG: Control Group; (ƞp^2^): Effect Size

## DISCUSSION

Our training intervention led to a significant improvement in creative thinking abilities and problem-solving skills, as evidenced by the differences observed between the experimental (TG) and control (CG) groups at post-test assessments. The nature of the RGs with complex rules likely contributed to this improvement. These games require players to think innovatively, adapt quickly, and utilize creative solutions to outwit opponents and maintain ball possession. By actively engaging in solving these complex problems during training sessions, the TG players enhanced their creative thinking abilities. In contrast, the CG, which did not undergo the training intervention, did not show any significant improvement in their creative thinking abilities, except for the originality of graphic-figural creativity, personal control of problem-solving inventory, and flexibility of verbal creativity indicex. This substantiates the assertion that soccer training involving complex rules in RGs may have been the influential factor leading to the observed improvements.

RGs with complex rules require players to constantly adapt and adjust their strategies based on the changing game situations [30]. This requires cognitive flexibility, which is the ability to switch between different ideas, perspectives, and strategies [31] By continuously engaging in these games, players develop a flexible mind-set that helps them think creatively and find innovative solutions [32, 33]. Complex rules in Rondo possession games introduce various challenges that players must overcome. They need to analyse the game situations, assess potential options, and make decisions quickly [34]. This process enhances their problem-solving abilities and encourages them to think outside the box. We found that the training intervention led to a significant improvement in the creative thinking abilities and problem-solving skills of U-13 soccer players. Specifically, the experimental group showed significant improvements in the level of Graphic-Figural Creativity, Problem-Solving Inventory, and Verbal Creativity scores. Therefore, it can be concluded that incorporating Rondo possession games with complex rules into training programmes can enhance the cognitive flexibility, problem-solving abilities, and creativity of U-13 soccer players.

Our intervention had a profound impact on the TG participants’ creative thinking abilities. This high-pressure decision-making enhances their ability to think creatively and make effective choices, even in stressful situations [35]. In RGs with complex rules, players are encouraged to take risks and experiment different techniques and strategies. This freedom allows them to express their creativity and try new approaches without fear of failure. By embracing risk-taking and self-expression, players develop confidence in their creative abilities, leading to more inventive thinking both on and off the field [36]. Complex rules in RGs often involve small playing areas, increasing the demand for spatial awareness and vision (visual scanning ability). Indeed, players must constantly analyse their surroundings, anticipate movements, and identify open spaces. This sharpening of spatial cognition enhances their ability to imagine possibilities, visualize alternative scenarios, and make creative decisions based on the game’s spatial dynamics [37].

We provide valuable insights into the psychological benefits of engaging in RGs. From a psychological perspective, problem solving is considered a critical cognitive skill that involves the ability to analyse situations, generate potential solutions, and make appropriate decisions in a short period of time and dynamically changing situations [38]. We demonstrated that practising Rondo possession games with complex rules can effectively enhance problem-solving abilities in U-13 youth soccer players. Research indicates that cognitive effort and engagement are crucial for skill development and problem solving [39]. By navigating the intricacies of the complex rules, players in the intervention group likely experienced higher cognitive engagement, leading to increased activation of problem-solving processes, which is important for their cognitive development towards adulthood [40]. The study implemented an 8-week training program, highlighting the importance of consistent and structured practice in skill development. This aligns with psychological research emphasizing deliberate practice as a key factor in becoming proficient in a particular skill [41]. The social and interactive nature of these games may contribute to the development of problem-solving skills. Rondo possession games typically involve multiple players working together towards a common goal [42]. This collaborative environment promotes social interaction, communication, and teamwork – all of which are important components of effective problem solving, potentially preparing for a more successful adulthood life.

Overall, we provide valuable evidence for the psychological advantages of engaging in Rondo possession games in improving problem-solving skills and creative thinking abilities for U-13 youth soccer players. It underscores the significance of cognitive flexibility, cognitive engagement, deliberate practice, and social interaction in the development of these skills. Further, based on these findings, it is recommended that coaches of U-13 soccer players incorporate the complex rules to improve cognitive skills during Rondo training, the study was conducted on U-13 soccer players, and the results may not be generalizable to other age groups or sports. Moreover, the study was conducted over an 8-week period, and the shorter-term effects of incorporating RGs with complex rules into training programmes are unknown.

For coaches and trainers, these insights offer a valuable perspective on youth soccer training. Incorporating such cognitive-rich exercises into regular training sessions can lead to the development of more tactically astute and versatile players. This approach could redefine youth soccer training paradigms, emphasizing not just physical prowess but also tactical intelligence and adaptability. Ultimately, this study demonstrates the potential of innovative training methodologies in shaping not only skilled athletes but also versatile and strategic thinkers on the soccer field. These findings underscore the importance of cognitive training in sports, particularly in young athletes who are adaptable, tactically aware, and prepared for the complexities of competitive soccer.

## CONCLUSIONS

In conclusion, we demonstrated that 8-week RGs training, incorporating varied geometric shapes, significantly enhanced problem solving, creative thinking, adaptability, and versatility in U-13 soccer players. This cognitive and strategic approach to training, which transcends traditional methods, equips young players with the skills to navigate the dynamic nature of soccer and excel in diverse playing positions.
